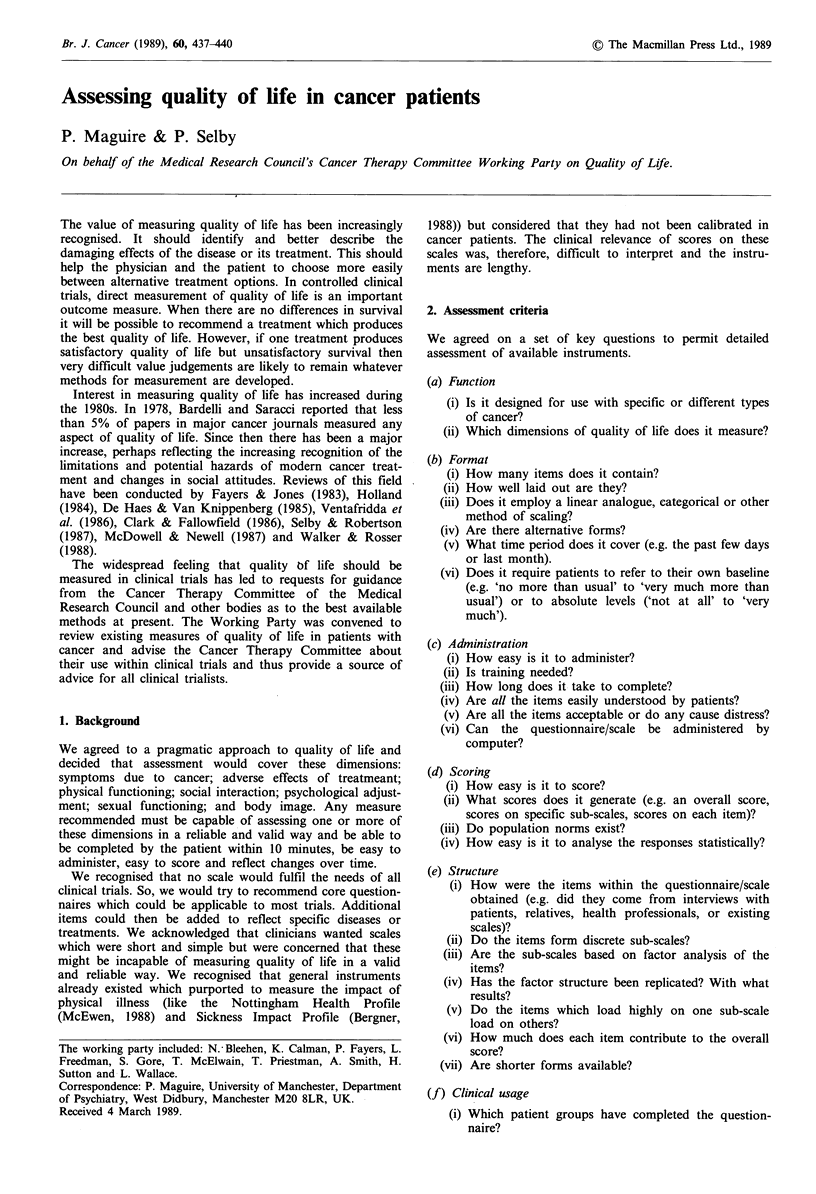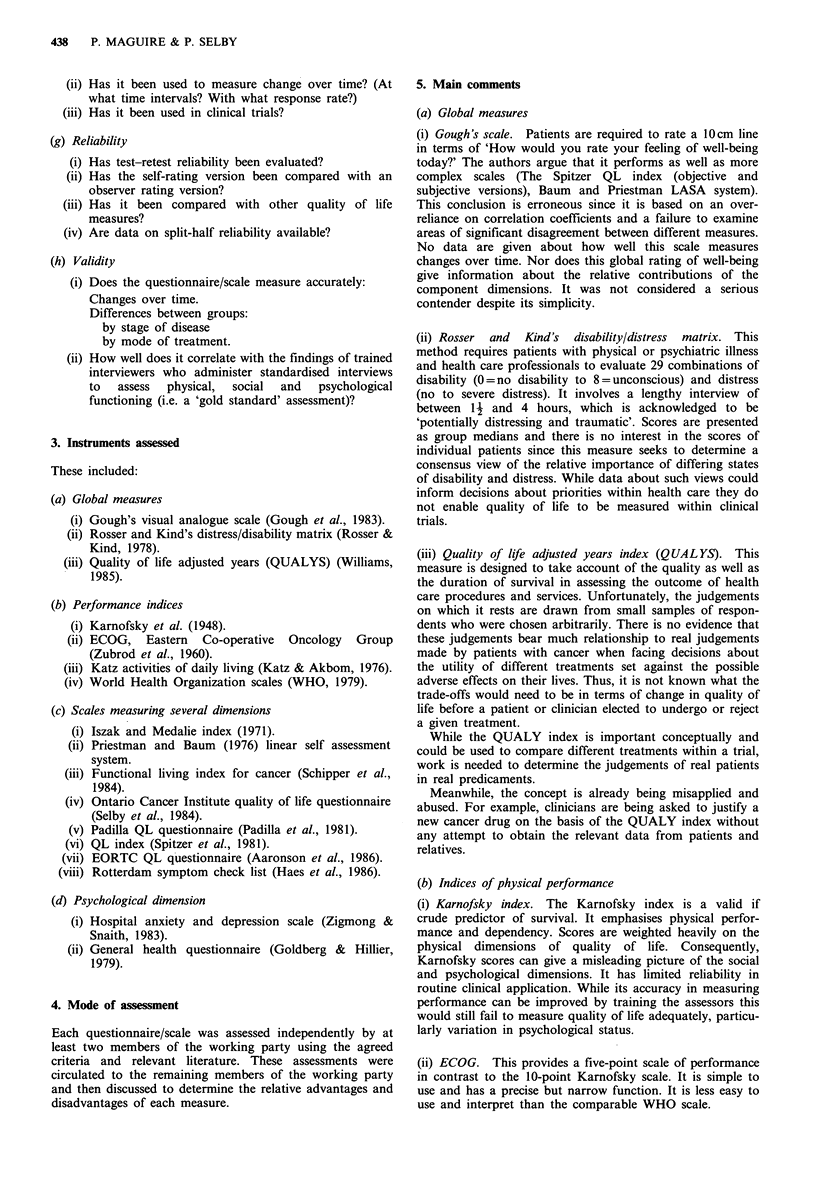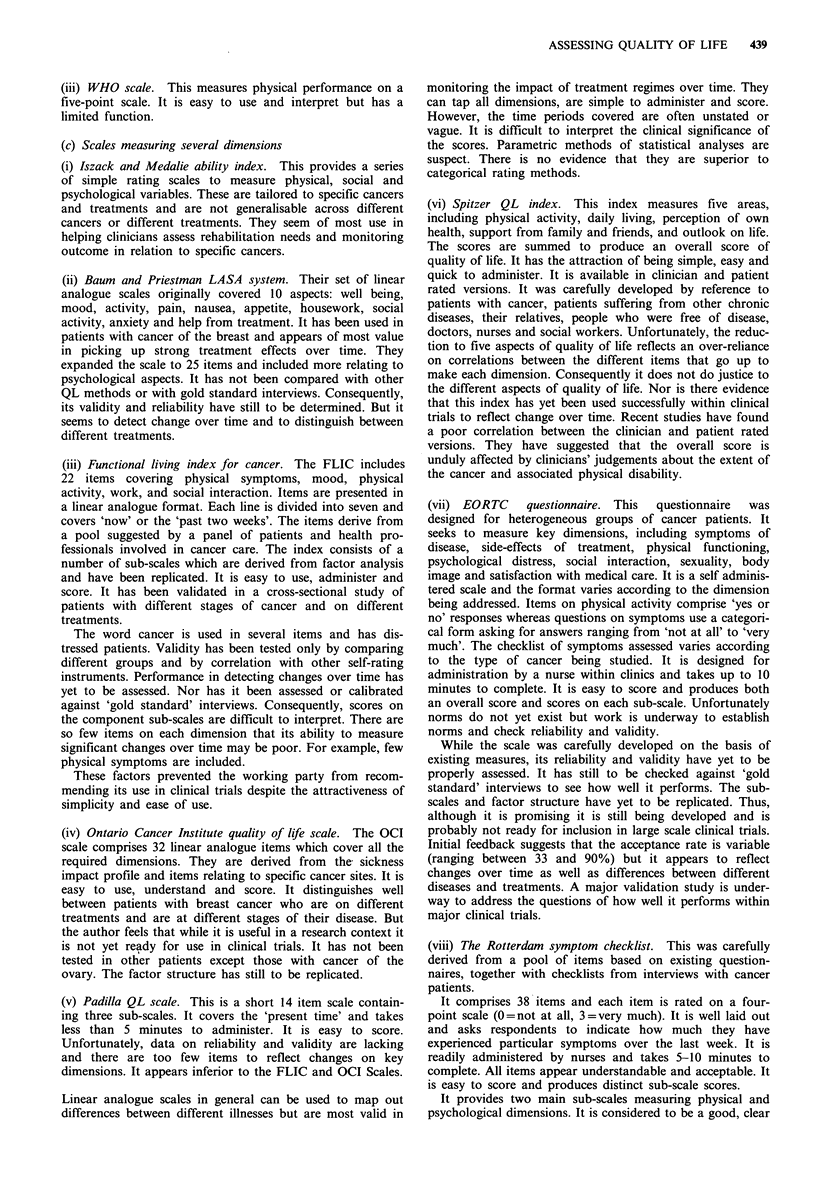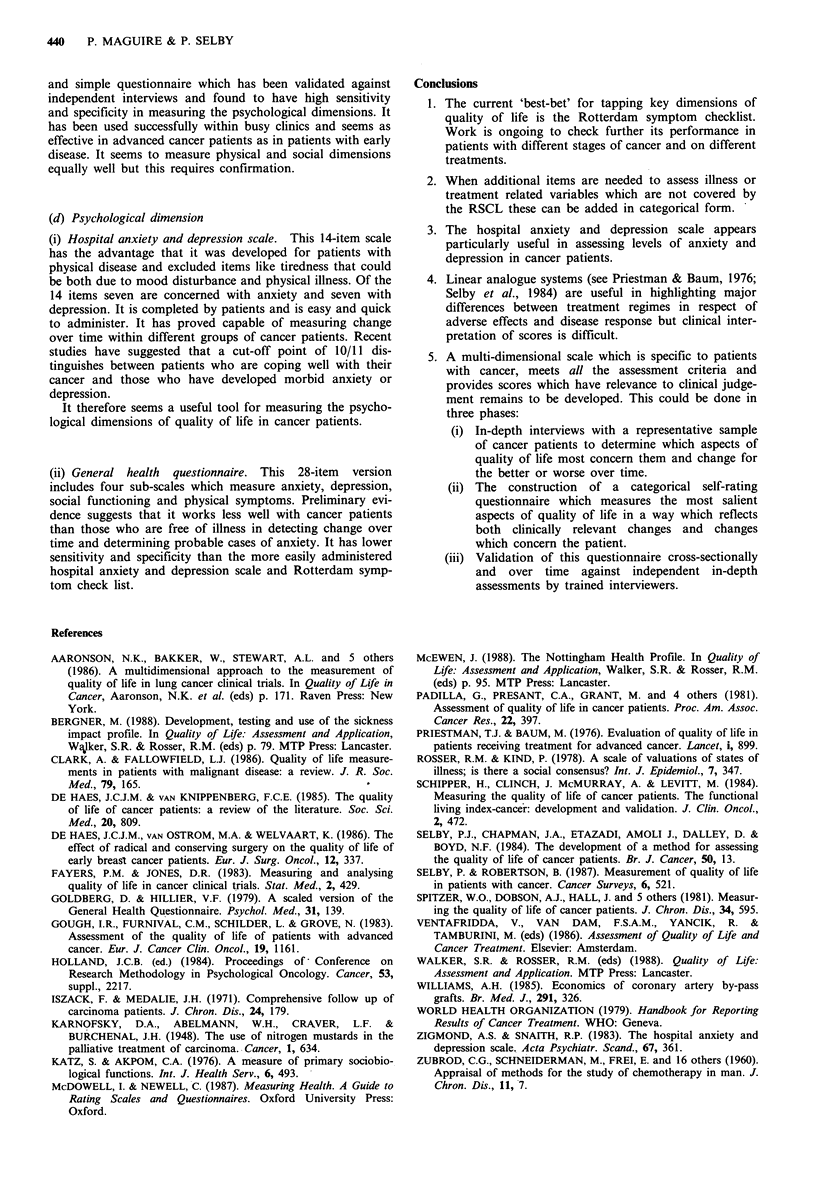# Assessing quality of life in cancer patients.

**DOI:** 10.1038/bjc.1989.301

**Published:** 1989-09

**Authors:** P. Maguire, P. Selby

**Affiliations:** Univ. of Manchester, Department of Psychiatry, UK.


Br. J. Cancer (1989), 60, 437–440 © The Macmillan Press Ltd., 1989

### Assessing quality of life in cancer patients

P. Maguire & P. Selby

*On behalf of the Medical Research Council's Cancer Therapy Committee Working Party on Quality of Life.*

The value of measuring quality of life has been increasingly recognised. It should identify and better describe the damaging effects of the disease or its treatment. This should help the physician and the patient to choose more easily between alternative treatment options. In controlled clinical trials, direct measurement of quality of life is an important outcome measure. When there are no differences in survival it will be possible to recommend a treatment which produces the best quality of life. However, if one treatment produces satisfactory quality of life but unsatisfactory survival then very difficult value judgements are likely to remain whatever methods for measurement are developed.

Interest in measuring quality of life has increased during the 1980s. In 1978, Bardelli and Saracci reported that less than 5% of papers in major cancer journals measured any aspect of quality of life. Since then there has been a major increase, perhaps reflecting the increasing recognition of the limitations and potential hazards of modern cancer treatment and changes in social attitudes. Reviews of this field have been conducted by Fayers & Jones (1983), Holland (1984), De Haes & Van Knippenberg (1985), Ventafridda *et al.* (1986), Clark & Fallowfield (1986), Selby & Robertson (1987), McDowell & Newell (1987) and Walker & Rosser (1988).

The widespread feeling that quality of life should be measured in clinical trials has led to requests for guidance from the Cancer Therapy Committee of the Medical Research Council and other bodies as to the best available methods at present. The Working Party was convened to review existing measures of quality of life in patients with cancer and advise the Cancer Therapy Committee about their use within clinical trials and thus provide a source of advice for all clinical trialists.

### 1. Background

We agreed to a pragmatic approach to quality of life and decided that assessment would cover these dimensions: symptoms due to cancer; adverse effects of treatmeant; physical functioning; social interaction; psychological adjustment; sexual functioning; and body image. Any measure recommended must be capable of assessing one or more of these dimensions in a reliable and valid way and be able to be completed by the patient within 10 minutes, be easy to administer, easy to score and reflect changes over time.

We recognised that no scale would fulfil the needs of all clinical trials. So, we would try to recommend core questionnaires which could be applicable to most trials. Additional items could then be added to reflect specific diseases or treatments. We acknowledged that clinicians wanted scales which were short and simple but were concerned that these might be incapable of measuring quality of life in a valid and reliable way. We recognised that general instruments already existed which purported to measure the impact of physical illness (like the Nottingham Health Profile (McEwen, 1988) and Sickness Impact Profile (Bergner, 1988)) but considered that they had not been calibrated in cancer patients. The clinical relevance of scores on these scales was, therefore, difficult to interpret and the instruments are lengthy.

The working party included: N. Bleehen, K. Calman, P. Fayers, L. Freedman, S. Gore, T. McElwain, T. Priestman, A. Smith, H. Sutton and L. Wallace.

Correspondence: P. Maguire, University of Manchester, Department of Psychiatry, West Didbury, Manchester M20 8LR, UK.

Received 4 March 1989.

### 2. Assessment criteria

We agreed on a set of key questions to permit detailed assessment of available instruments.

(*a*) *Function*

(i) Is it designed for use with specific or different types of cancer?
(ii) Which dimensions of quality of life does it measure?

(*b*) *Format*

(i) How many items does it contain?
(ii) How well laid out are they?
(iii) Does it employ a linear analogue, categorical or other method of scaling?
(iv) Are there alternative forms?
(v) What time period does it cover (e.g. the past few days or last month).
(vi) Does it require patients to refer to their own baseline (e.g. 'no more than usual' to 'very much more than usual') or to absolute levels ('not at all' to 'very much').

(*c*) *Administration*

(i) How easy is it to administer?
(ii) Is training needed?
(iii) How long does it take to complete?
(iv) Are *all* the items easily understood by patients?
(v) Are all the items acceptable or do any cause distress?
(vi) Can the questionnaire/scale be administered by computer?

(*d*) *Scoring*

(i) How easy is it to score?
(ii) What scores does it generate (e.g. an overall score, scores on specific sub-scales, scores on each item)?
(iii) Do population norms exist?
(iv) How easy is it to analyse the responses statistically?

(*e*) *Structure*

(i) How were the items within the questionnaire/scale obtained (e.g. did they come from interviews with patients, relatives, health professionals, or existing scales)?
(ii) Do the items form discrete sub-scales?
(iii) Are the sub-scales based on factor analysis of the items?
(iv) Has the factor structure been replicated? With what results?
(v) Do the items which load highly on one sub-scale load on others?
(vi) How much does each item contribute to the overall score?
(vii) Are shorter forms available?

(*f*) *Clinical usage*

(i) Which patient groups have completed the questionnaire?

(ii) Has it been used to measure change over time? (At what time intervals? With what response rate?)
(iii) Has it been used in clinical trials?

(g) *Reliability*

(i) Has test–retest reliability been evaluated?
(ii) Has the self-rating version been compared with an observer rating version?
(iii) Has it been compared with other quality of life measures?
(iv) Are data on split-half reliability available?

(h) *Validity*

(i) Does the questionnaire/scale measure accurately:
Changes over time.
Differences between groups:
by stage of disease
by mode of treatment.
(ii) How well does it correlate with the findings of trained interviewers who administer standardised interviews to assess physical, social and psychological functioning (i.e. a 'gold standard' assessment)?

### 3. Instruments assessed

These included:

(a) *Global measures*

(i) Gough's visual analogue scale (Gough *et al*., 1983).
(ii) Rosser and Kind's distress/disability matrix (Rosser & Kind, 1978).
(iii) Quality of life adjusted years (QUALYS) (Williams, 1985).

(b) *Performance indices*

(i) Karnofsky *et al.* (1948).
(ii) ECOG, Eastern Co-operative Oncology Group (Zubrod *et al*., 1960).
(iii) Katz activities of daily living (Katz & Akbom, 1976).
(iv) World Health Organization scales (WHO, 1979).

(c) *Scales measuring several dimensions*

(i) Iszak and Medalie index (1971).
(ii) Priestman and Baum (1976) linear self assessment system.
(iii) Functional living index for cancer (Schipper *et al*., 1984).
(iv) Ontario Cancer Institute quality of life questionnaire (Selby *et al*., 1984).
(v) Padilla QL questionnaire (Padilla *et al*., 1981).
(vi) QL index (Spitzer *et al*., 1981).
(vii) EORTC QL questionnaire (Aaronson *et al*., 1986).
(viii) Rotterdam symptom check list (Haes *et al*., 1986).

(d) *Psychological dimension*

(i) Hospital anxiety and depression scale (Zigmong & Snaith, 1983).
(ii) General health questionnaire (Goldberg & Hillier, 1979).

### 4. Mode of assessment

Each questionnaire/scale was assessed independently by at least two members of the working party using the agreed criteria and relevant literature. These assessments were circulated to the remaining members of the working party and then discussed to determine the relative advantages and disadvantages of each measure.

### 5. Main comments

(a) *Global measures*

(i) *Gough's scale*. Patients are required to rate a 10 cm line in terms of 'How would you rate your feeling of well-being today?' The authors argue that it performs as well as more complex scales (The Spitzer QL index (objective and subjective versions), Baum and Priestman LASA system). This conclusion is erroneous since it is based on an over-reliance on correlation coefficients and a failure to examine areas of significant disagreement between different measures. No data are given about how well this scale measures changes over time. Nor does this global rating of well-being give information about the relative contributions of the component dimensions. It was not considered a serious contender despite its simplicity.

(ii) *Rosser and Kind's disability/distress matrix*. This method requires patients with physical or psychiatric illness and health care professionals to evaluate 29 combinations of disability (0 = no disability to 8 = unconscious) and distress (no to severe distress). It involves a lengthy interview of between 1½ and 4 hours, which is acknowledged to be 'potentially distressing and traumatic'. Scores are presented as group medians and there is no interest in the scores of individual patients since this measure seeks to determine a consensus view of the relative importance of differing states of disability and distress. While data about such views could inform decisions about priorities within health care they do not enable quality of life to be measured within clinical trials.

(iii) *Quality of life adjusted years index* (*QUALYS*). This measure is designed to take account of the quality as well as the duration of survival in assessing the outcome of health care procedures and services. Unfortunately, the judgements on which it rests are drawn from small samples of respondents who were chosen arbitrarily. There is no evidence that these judgements bear much relationship to real judgements made by patients with cancer when facing decisions about the utility of different treatments set against the possible adverse effects on their lives. Thus, it is not known what the trade-offs would need to be in terms of change in quality of life before a patient or clinician elected to undergo or reject a given treatment.

While the QUALY index is important conceptually and could be used to compare different treatments within a trial, work is needed to determine the judgements of real patients in real predicaments.

Meanwhile, the concept is already being misapplied and abused. For example, clinicians are being asked to justify a new cancer drug on the basis of the QUALY index without any attempt to obtain the relevant data from patients and relatives.

(b) *Indices of physical performance*

(i) *Karnofsky index*. The Karnofsky index is a valid if crude predictor of survival. It emphasises physical performance and dependency. Scores are weighted heavily on the physical dimensions of quality of life. Consequently, Karnofsky scores can give a misleading picture of the social and psychological dimensions. It has limited reliability in routine clinical application. While its accuracy in measuring performance can be improved by training the assessors this would still fail to measure quality of life adequately, particularly variation in psychological status.

(ii) *ECOG*. This provides a five-point scale of performance in contrast to the 10-point Karnofsky scale. It is simple to use and has a precise but narrow function. It is less easy to use and interpret than the comparable WHO scale.

(iii) *WHO scale.* This measures physical performance on a five-point scale. It is easy to use and interpret but has a limited function.

(*c*) *Scales measuring several dimensions*

(i) *Iszack and Medalie ability index.* This provides a series of simple rating scales to measure physical, social and psychological variables. These are tailored to specific cancers and treatments and are not generalisable across different cancers or different treatments. They seem of most use in helping clinicians assess rehabilitation needs and monitoring outcome in relation to specific cancers.

(ii) *Baum and Priestman LASA system.* Their set of linear analogue scales originally covered 10 aspects: well being, mood, activity, pain, nausea, appetite, housework, social activity, anxiety and help from treatment. It has been used in patients with cancer of the breast and appears of most value in picking up strong treatment effects over time. They expanded the scale to 25 items and included more relating to psychological aspects. It has not been compared with other QL methods or with gold standard interviews. Consequently, its validity and reliability have still to be determined. But it seems to detect change over time and to distinguish between different treatments.

(iii) *Functional living index for cancer.* The FLIC includes 22 items covering physical symptoms, mood, physical activity, work, and social interaction. Items are presented in a linear analogue format. Each line is divided into seven and covers 'now' or the 'past two weeks'. The items derive from a pool suggested by a panel of patients and health professionals involved in cancer care. The index consists of a number of sub-scales which are derived from factor analysis and have been replicated. It is easy to use, administer and score. It has been validated in a cross-sectional study of patients with different stages of cancer and on different treatments.

The word cancer is used in several items and has distressed patients. Validity has been tested only by comparing different groups and by correlation with other self-rating instruments. Performance in detecting changes over time has yet to be assessed. Nor has it been assessed or calibrated against 'gold standard' interviews. Consequently, scores on the component sub-scales are difficult to interpret. There are so few items on each dimension that its ability to measure significant changes over time may be poor. For example, few physical symptoms are included.

These factors prevented the working party from recommending its use in clinical trials despite the attractiveness of simplicity and ease of use.

(iv) *Ontario Cancer Institute quality of life scale.* The OCI scale comprises 32 linear analogue items which cover all the required dimensions. They are derived from the sickness impact profile and items relating to specific cancer sites. It is easy to use, understand and score. It distinguishes well between patients with breast cancer who are on different treatments and are at different stages of their disease. But the author feels that while it is useful in a research context it is not yet ready for use in clinical trials. It has not been tested in other patients except those with cancer of the ovary. The factor structure has still to be replicated.

(v) *Padilla QL scale.* This is a short 14 item scale containing three sub-scales. It covers the 'present time' and takes less than 5 minutes to administer. It is easy to score. Unfortunately, data on reliability and validity are lacking and there are too few items to reflect changes on key dimensions. It appears inferior to the FLIC and OCI Scales.

Linear analogue scales in general can be used to map out differences between different illnesses but are most valid in monitoring the impact of treatment regimes over time. They can tap all dimensions, are simple to administer and score. However, the time periods covered are often unstated or vague. It is difficult to interpret the clinical significance of the scores. Parametric methods of statistical analyses are suspect. There is no evidence that they are superior to categorical rating methods.

(vi) *Spitzer QL index.* This index measures five areas, including physical activity, daily living, perception of own health, support from family and friends, and outlook on life. The scores are summed to produce an overall score of quality of life. It has the attraction of being simple, easy and quick to administer. It is available in clinician and patient rated versions. It was carefully developed by reference to patients with cancer, patients suffering from other chronic diseases, their relatives, people who were free of disease, doctors, nurses and social workers. Unfortunately, the reduction to five aspects of quality of life reflects an over-reliance on correlations between the different items that go up to make each dimension. Consequently it does not do justice to the different aspects of quality of life. Nor is there evidence that this index has yet been used successfully within clinical trials to reflect change over time. Recent studies have found a poor correlation between the clinician and patient rated versions. They have suggested that the overall score is unduly affected by clinicians' judgements about the extent of the cancer and associated physical disability.

(vii) *EORTC questionnaire.* This questionnaire was designed for heterogeneous groups of cancer patients. It seeks to measure key dimensions, including symptoms of disease, side-effects of treatment, physical functioning, psychological distress, social interaction, sexuality, body image and satisfaction with medical care. It is a self administered scale and the format varies according to the dimension being addressed. Items on physical activity comprise 'yes or no' responses whereas questions on symptoms use a categorical form asking for answers ranging from 'not at all' to 'very much'. The checklist of symptoms assessed varies according to the type of cancer being studied. It is designed for administration by a nurse within clinics and takes up to 10 minutes to complete. It is easy to score and produces both an overall score and scores on each sub-scale. Unfortunately norms do not yet exist but work is underway to establish norms and check reliability and validity.

While the scale was carefully developed on the basis of existing measures, its reliability and validity have yet to be properly assessed. It has still to be checked against 'gold standard' interviews to see how well it performs. The sub-scales and factor structure have yet to be replicated. Thus, although it is promising it is still being developed and is probably not ready for inclusion in large scale clinical trials. Initial feedback suggests that the acceptance rate is variable (ranging between 33 and 90%) but it appears to reflect changes over time as well as differences between different diseases and treatments. A major validation study is underway to address the questions of how well it performs within major clinical trials.

(viii) *The Rotterdam symptom checklist.* This was carefully derived from a pool of items based on existing questionnaires, together with checklists from interviews with cancer patients.

It comprises 38 items and each item is rated on a four-point scale (0 = not at all, 3 = very much). It is well laid out and asks respondents to indicate how much they have experienced particular symptoms over the last week. It is readily administered by nurses and takes 5–10 minutes to complete. All items appear understandable and acceptable. It is easy to score and produces distinct sub-scale scores.

It provides two main sub-scales measuring physical and psychological dimensions. It is considered to be a good, clear and simple questionnaire which has been validated against independent interviews and found to have high sensitivity and specificity in measuring the psychological dimensions. It has been used successfully within busy clinics and seems as effective in advanced cancer patients as in patients with early disease. It seems to measure physical and social dimensions equally well but this requires confirmation.

(*d*) *Psychological dimension*

(i) *Hospital anxiety and depression scale*. This 14-item scale has the advantage that it was developed for patients with physical disease and excluded items like tiredness that could be both due to mood disturbance and physical illness. Of the 14 items seven are concerned with anxiety and seven with depression. It is completed by patients and is easy and quick to administer. It has proved capable of measuring change over time within different groups of cancer patients. Recent studies have suggested that a cut-off point of 10/11 distinguishes between patients who are coping well with their cancer and those who have developed morbid anxiety or depression.

It therefore seems a useful tool for measuring the psychological dimensions of quality of life in cancer patients.

(ii) *General health questionnaire*. This 28-item version includes four sub-scales which measure anxiety, depression, social functioning and physical symptoms. Preliminary evidence suggests that it works less well with cancer patients than those who are free of illness in detecting change over time and determining probable cases of anxiety. It has lower sensitivity and specificity than the more easily administered hospital anxiety and depression scale and Rotterdam symptom check list.

### Conclusions

1. The current 'best-bet' for tapping key dimensions of quality of life is the Rotterdam symptom checklist. Work is ongoing to check further its performance in patients with different stages of cancer and on different treatments.
2. When additional items are needed to assess illness or treatment related variables which are not covered by the RSCL these can be added in categorical form.
3. The hospital anxiety and depression scale appears particularly useful in assessing levels of anxiety and depression in cancer patients.
4. Linear analogue systems (see Priestman & Baum, 1976; Selby *et al.*, 1984) are useful in highlighting major differences between treatment regimes in respect of adverse effects and disease response but clinical interpretation of scores is difficult.
5. A multi-dimensional scale which is specific to patients with cancer, meets *all* the assessment criteria and provides scores which have relevance to clinical judgement remains to be developed. This could be done in three phases:
   (i) In-depth interviews with a representative sample of cancer patients to determine which aspects of quality of life most concern them and change for the better or worse over time.
   (ii) The construction of a categorical self-rating questionnaire which measures the most salient aspects of quality of life in a way which reflects both clinically relevant changes and changes which concern the patient.
   (iii) Validation of this questionnaire cross-sectionally and over time against independent in-depth assessments by trained interviewers.

### References

AARONSON, N.K., BAKKER, W., STEWART, A.L. and 5 others (1986). A multidimensional approach to the measurement of quality of life in lung cancer clinical trials. In *Quality of Life in Cancer*, Aaronson, N.K. *et al.* (eds) p. 171. Raven Press: New York.

BERGNER, M. (1988). Development, testing and use of the sickness impact profile. In *Quality of Life: Assessment and Application*, Walker, S.R. & Rosser, R.M. (eds) p. 79. MTP Press: Lancaster.

CLARK, A. & FALLOWFIELD, L.J. (1986). Quality of life measurements in patients with malignant disease: a review. *J. R. Soc. Med.*, **79**, 165.

DE HAES, J.C.J.M. & VAN KNIPPENBERG, F.C.E. (1985). The quality of life of cancer patients: a review of the literature. *Soc. Sci. Med.*, **20**, 809.

DE HAES, J.C.J.M., VAN OSTROM, M.A. & WELVAART, K. (1986). The effect of radical and conserving surgery on the quality of life of early breast cancer patients. *Eur. J. Surg. Oncol.*, **12**, 337.

FAYERS, P.M. & JONES, D.R. (1983). Measuring and analysing quality of life in cancer clinical trials. *Stat. Med.*, **2**, 429.

GOLDBERG, D. & HILLIER, V.F. (1979). A scaled version of the General Health Questionnaire. *Psychol. Med.*, **31**, 139.

GOUGH, I.R., FURNIVAL, C.M., SCHILDER, L. & GROVE, N. (1983). Assessment of the quality of life of patients with advanced cancer. *Eur. J. Cancer Clin. Oncol.*, **19**, 1161.

HOLLAND, J.C.B. (ed.) (1984). Proceedings of Conference on Research Methodology in Psychological Oncology. *Cancer*, **53**, suppl., 2217.

ISZACK, F. & MEDALIE, J.H. (1971). Comprehensive follow up of carcinoma patients. *J. Chron. Dis.*, **24**, 179.

KARNOFSKY, D.A., ABELMANN, W.H., CRAVER, L.F. & BURCHENAL, J.H. (1948). The use of nitrogen mustards in the palliative treatment of carcinoma. *Cancer*, **1**, 634.

KATZ, S. & AKPOM, C.A. (1976). A measure of primary sociobiological functions. *Int. J. Health Serv.*, **6**, 493.

McDOWELL, I. & NEWELL, C. (1987). *Measuring Health. A Guide to Rating Scales and Questionnaires.* Oxford University Press: Oxford.

McEWEN, J. (1988). The Nottingham Health Profile. In *Quality of Life: Assessment and Application*, Walker, S.R. & Rosser, R.M. (eds) p. 95. MTP Press: Lancaster.

PADILLA, G., PRESANT, C.A., GRANT, M. and 4 others (1981). Assessment of quality of life in cancer patients. *Proc. Am. Assoc. Cancer Res.*, **22**, 397.

PRIESTMAN, T.J. & BAUM, M. (1976). Evaluation of quality of life in patients receiving treatment for advanced cancer. *Lancet*, **i**, 899.

ROSSER, R.M. & KIND, P. (1978). A scale of valuations of states of illness; is there a social consensus? *Int. J. Epidemiol.*, **7**, 347.

SCHIPPER, H., CLINCH, J. McMURRAY, A. & LEVITT, M. (1984). Measuring the quality of life of cancer patients. The functional living index-cancer: development and validation. *J. Clin. Oncol.*, **2**, 472.

SELBY, P.J., CHAPMAN, J.A., ETAZADI, AMOLI J., DALLEY, D. & BOYD, N.F. (1984). The development of a method for assessing the quality of life of cancer patients. *Br. J. Cancer*, **50**, 13.

SELBY, P. & ROBERTSON, B. (1987). Measurement of quality of life in patients with cancer. *Cancer Surveys*, **6**, 521.

SPITZER, W.O., DOBSON, A.J., HALL, J. and 5 others (1981). Measuring the quality of life of cancer patients. *J. Chron. Dis.*, **34**, 595.

VENTAFRIDDA, V., VAN DAM, F.S.A.M., YANCIK, R. & TAMBURINI, M. (eds) (1986). *Assessment of Quality of Life and Cancer Treatment.* Elsevier: Amsterdam.

WALKER, S.R. & ROSSER, R.M. (eds) (1988). *Quality of Life: Assessment and Application.* MTP Press: Lancaster.

WILLIAMS, A.H. (1985). Economics of coronary artery by-pass grafts. *Br. Med. J.*, **291**, 326.

WORLD HEALTH ORGANIZATION (1979). *Handbook for Reporting Results of Cancer Treatment.* WHO: Geneva.

ZIGMOND, A.S. & SNAITH, R.P. (1983). The hospital anxiety and depression scale. *Acta Psychiatr. Scand.*, **67**, 361.

ZUBROD, C.G., SCHNEIDERMAN, M., FREI, E. and 16 others (1960). Appraisal of methods for the study of chemotherapy in man. *J. Chron. Dis.*, **11**, 7.